# The Use of Sixty Degree Rotation of the Acetabulum for Treatment of Dogs With Canine Hip Dysplasia. A Short Case Series

**DOI:** 10.3389/fvets.2021.669680

**Published:** 2021-05-13

**Authors:** Horia Elefterescu, Ciprian Andrei Ober, Andrei Timen, Christos Yiapanis, William McCartney, Joshua Milgram

**Affiliations:** ^1^Clinica de Chirurgie si Ortopedie Veterinara, Bucuresti, Romania; ^2^Department of Surgery, Faculty of Veterinary Medicine, University of Agricultural Sciences and Veterinary Medicine, Cluj-Napoca, Romania; ^3^Trivet, Cluj-Napoca, Romania; ^4^Cyvets Veterinary Centre, Paphos, Cyprus; ^5^North Dublin Orthopaedic Animal Hospital, Dublin, Ireland; ^6^Department of Small Animal Surgery, The Robert H. Smith Faculty of Agriculture, Food and Environment, Koret School of Veterinary Medicine, The Hebrew University of Jerusalem, Rehovot, Israel

**Keywords:** dog, hip dysplasia, femoral head coverage, osteoarthritis, radiography

## Abstract

Triple pelvic osteotomy (TPO) is a prophylactic surgical procedure performed on dogs with canine hip dysplasia. The procedure is indicated in skeletally immature dogs without secondary osteoarthritis (OA). It has been suggested that 60° of rotation is excessive and is associated with poor outcome. The objective of the study was to assess the medium term outcome in dogs having undergone triple pelvic osteotomy (TPO) using 60° dedicated plates. Nine TPOs were performed in seven dogs with hip dysplasia. Eight of nine hips had 72–100% osseous union at the time of revisit. The mean time to final radiographic recheck was 200 days (range, 185–229 days). The mean time to follow-up was 11.5 months (range 11–12 months). All 7 dogs had regained full function and did not require supplemental analgesia. Pelvic canal narrowing was noted in the two dogs with bilateral surgeries, but no clinical consequences were noted according to owner's statement.If more than 40 degrees reduction angles at Ortolani test, 60° of rotation of the acetabulum can be used successfully in dogs with hip dysplasia. At the time of mid-term follow-up, all dogs in this case series had full function.

## Introduction

Triple pelvic osteotomy (TPO) is a prophylactic surgical procedure which changes the position of the acetabulum to improve the biomechanics of the hip joint. The axial rotation and lateralization of the dorsal acetabular rim achieved by TPO provides greater coverage of the femoral head by the acetabulum, improving joint congruence and reducing secondary degenerative changes associated with hip dysplasia (1).

Biomechanical studies have shown that triple pelvic osteotomy can also reduce the magnitude of the force acting on the load-bearing portions of the acetabular rim and the femoral head and increase the contact area on which the force acts (1, 2). Successful clinical outcomes have been reported despite development of osteoarthritis in some studies; however, it is unknown to what degree the osteotomy retarded the progression of osteoarthritis (3, 4).

According to some authors, rotation beyond 40 degrees is not advised because it is unlikely to yield further improved coverage of the femoral head, it will worsen pelvic canal narrowing, it may result in impingement of the dorsal acetabular rim on the femoral neck, and dogs requiring more extreme rotation are not likely good candidates for the procedure (5).

The purpose of this study was to confirm based on radiographic and clinical outcomes, that the rotation of 60° is a viable option for dogs in which standard rotation angles are inadequate and it has no clinical consequences because of worsening of the pelvic canal narrowing.

## Materials and Methods

### Data Collection

**Figure 1:**
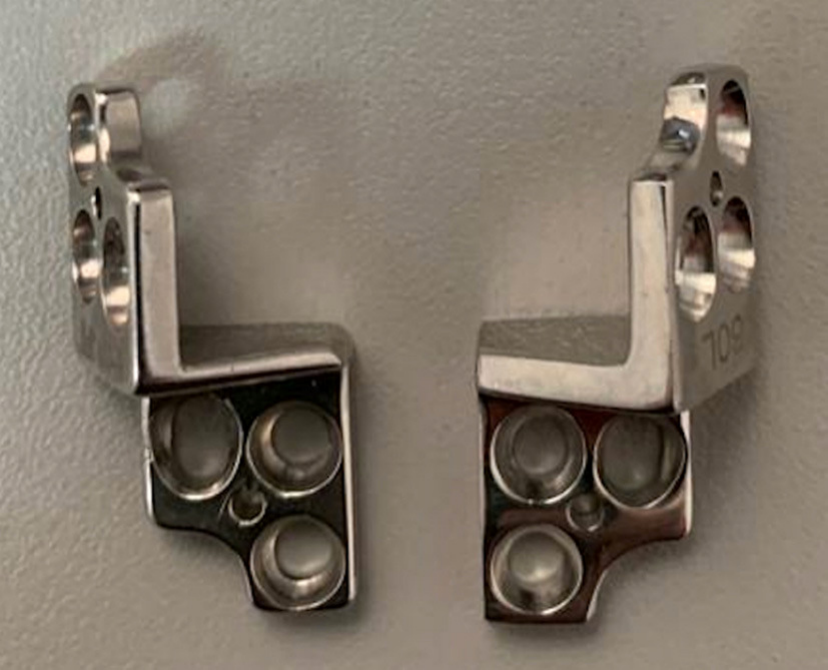
Plate used for pelvic osteotomies (Courtesy M/S Pakrom International, Sialkot, Pakistan).

**Figure 2:**
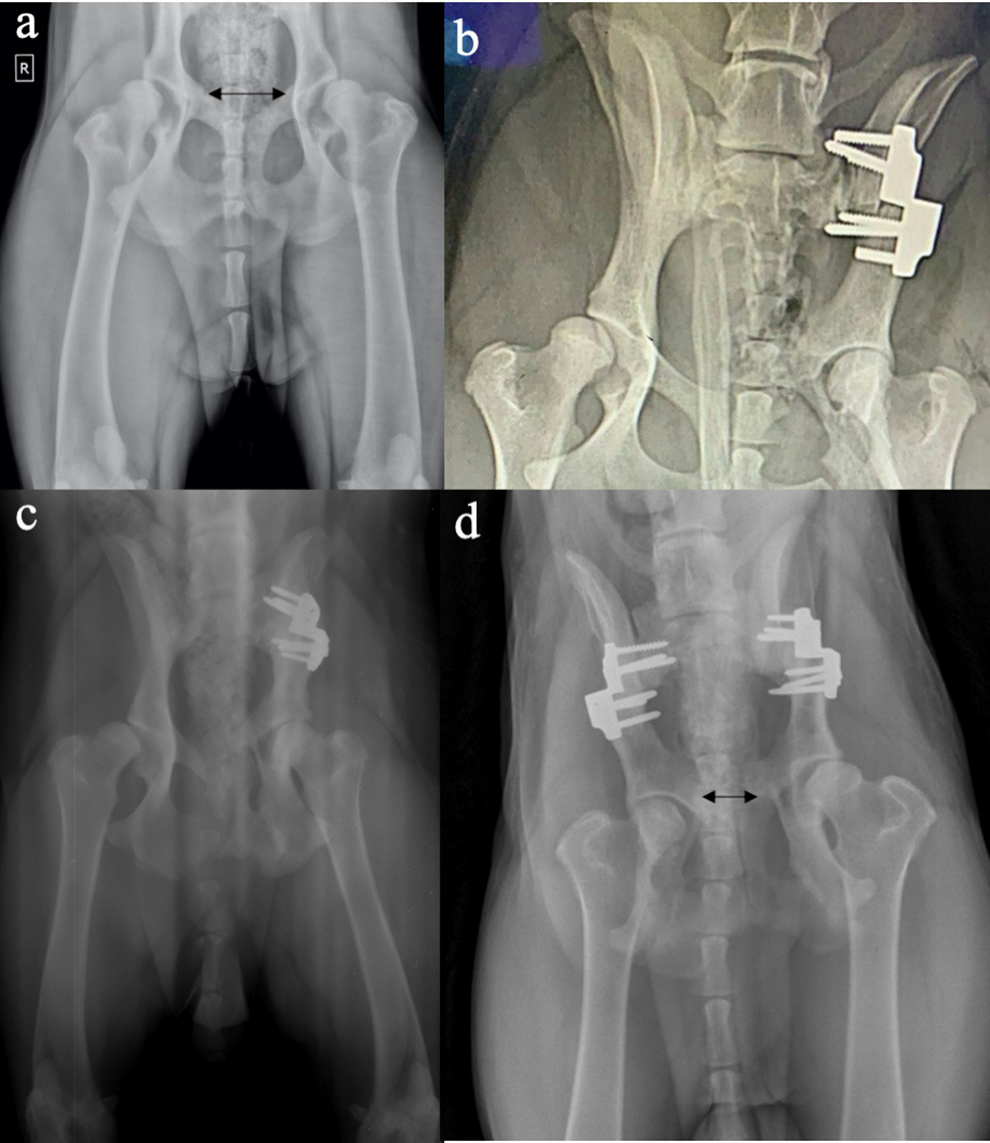
Ventrodorsal radiographs of the hip joints before **(a)**, and immediately after **(b)**, and at 6 months (unilateral) **(c)** and bilateral **(d)** triple pelvic osteotomy. Note the improved congruence and acetabular support on the postoperative radiographs. Also note the difference in the distance between the lateral edges of the pubic osteotomies (double-headed arrows) pre- and postoperative, suggesting 40% of pelvic canal narrowing with the triple pelvic osteotomy.

Medical records were reviewed to identify dogs that had early stages of hip dysplasia treated with a TPO surgery. Criteria for inclusion were: osteotomy using a dedicated TPO 60°plate (M/S Pakrom International, Sialkot, Pakistan) (Figure 1), pre- and immediate postoperative radiographs, short term (3–6 months) and mid-term (6–12 months) radiographs and clinical evaluation and email interview based on validated questions at a minimum of 6 months (6–12 months). All the dogs' owners completed the questionnaire items. Total hip replacement (THR) was not an option because of financial reasons. In addition, all cases needed to have preoperative (Figure 2a) and immediate postoperative ventrodorsal (Figures 2b,c) and mediolateral pelvic radiographs available for review. Data derived from medical records included breed, sex, site of surgery, age, body weight at time of surgery, perioperative or postoperative complications.

### Radiographic and Clinical Assessment

Ortolani testing was performed in all dogs under sedation. HE and CAO performed the Ortolani tests and angles of reduction of more than 40 degrees in all dogs were subjectively assessed. Ventrodorsal radiographs taken at least 6 months post-operatively were used to evaluate osteotomy healing with a previously described 5-point grading system (4 = 76–100%; 3 = 51–75%; 2 = 26–50%; 15 = −25%; and 0 = no osseous bridging (6, 7).

### Surgical Technique

The dogs were administered acepromazine (0.025 mg/kg, IM) and hydromorphone (0, 1 mg/kg, IM) as premedication. The induction was made with propofol (3–6 mg/kg, IV) and maintained with isoflurane and oxygen. Under general anesthesia, an epidural with preservative-free morphine (0.1 mg/kg) was given as an adjunct analgesic. The patients were positioned in lateral recumbency, with the dorsal plane perpendicular to the table. The pubic osteotomy (or ostectomy) was performed first, with the pelvic limb held in abduction. A large section of pubis was excised (about 1 cm) using a 5 mm Rongeur according to guidelines for the procedure (5). The ischial osteotomy was performed second using a Gigli wire. The lateral approach to the ilium was via the gluteal “roll-up” technique (8). The osteotomy was performed perpendicular to the long axis of the pelvis using an oscillating saw. Plate fixation was performed using specialty 60° non-locking plates made specifically for this procedure (Figure 1).

### Preoperative and Postoperative Care

Cefazolin (22 mg/kg, IV) was given 30 min before induction and every 90 min perioperatively. Tramadol (3–5 mg/kg orally every 6 h) and a non-steroidal anti-inflammatory drug (deracoxib [2 mg/kg orally daily]) were prescribed for continued analgesia at home for 10 days. Cephalexin (25 mg/kg orally every 12 h) was also given to continue antibiotics for 7 more days to help prevent infection. When home, supervised activity (no running, playing or jumping) was recommended for 6–8 weeks.

### Mid-Term Follow-Up

Follow-up was conducted by completing a visual analog scale questionnaire (9) during a telephone or e-mail interview with owners (Table 2). The questionnaire consisted of 7 questions that adhered to the format of a 10 cm VAS (9). Some of the questions were in the following format: What is willing to play voluntarily? Is stiffness at the beginning of the day present? There was a corresponding VAS line for each question with contrasting descriptors at each end (e.g., very good willingness to play voluntarily or no stiffness at the beginning of the day, respectively). Suture removal was performed at 2 weeks. Owners were queried regarding their dog's function after surgery. Clinically, the cases were categorized into 1 of 3 clinical outcomes: full function, acceptable function, or unacceptable function as defined by Cook et al. (2010) (10). Full function was defined as restoration to full intended level and duration of activities. Acceptable function was defined as restoration to intended activities and performance from preinjury that was limited in level or duration and/or requires medication to achieve. Unacceptable function was defined as all other outcomes. Final orthopedic exams including Ortolani test were performed between 11 and 12 months postoperative for all dogs.

### Complications

Intraoperative or short-term postoperative complications that were noted in the medical records or on postoperative radiographs. Complications were identified as catastrophic, major, and minor. Catastrophic was considered any complication that caused permanent unacceptable function, was directly related to death, or was the cause for euthanasia. Major complications required medical or surgical treatment. Minor complications were considered those that did not require any additional surgery or medication (10). Owners were also questioned relative to the occurrence of complications after final veterinarian evaluation.

## Results

Nine TPO procedures were performed in seven dogs. Two dogs had staged, bilateral TPOs performed 4–5 weeks apart. Breeds were identified as Labrador (2), American Bulldog (1), German Shepherd (1), Cane Corso (1), Central Asian Shepherd (1) and mixed breed (1). The population included 4 males and 3 females, median age 12 months and median bodyweight 37 kg (Table 1). No dogs revealed osteophyte formation before surgical treatment. Ortolani sign was combined with complete assessment of the patient, and subjectively we assessed more than 40 degrees reduction angles.

**Table 1:** Summary statistics for breeds, sex, site of surgery, age at surgery (month), body weight (kg), follow-up (day).

**Case no**	**Breed**	**Sex**	**Site of surgery**	**Age at surgery (month)**	**Body weight (kg)**	**Follow-up (day)^a^**
1	American Bulldog	F	left	24	44	189
2	German Shepherd	M	bilateral	10, 12^b^	32, 37^c^	223
3	Cane Corso	F	left	8	36	191
4	Labrador	M	right	12	43	185
5	Central Asian Shepherd	F	left	17	52	198
6	Mixed breed	M	right	8	20	188
7	Labrador	M	bilateral	7, 9^b^	31, 36^c^	229

a *Duration means the interval between the pre- and the postoperative radiographs. In case of bilateral procedure, the duration number means the interval between the preoperative radiograph and the last radiographic follow-up*.

b *Age at the first and at the second surgery*.

c *Body weight at the first and at the second surgery*.

### Postoperative Radiographic Assessment

Eight of nine hips had 72–100% osseous union at the time of revisit. The mean time to final radiographic recheck was 200 days (range, 185–229 days). After surgery, craniodorsal coverage of the femoral head was increased (Figures 2b–d) in all the operated hips. There was no evidence of radiographic progression of degenerative joint disease at final radiographic assessment.

### Mid-Term Follow-Up

The mean time to final clinical follow-up was 11.5 months (range 11–12 months). All dogs were alive and available for follow-up. All owners were satisfied with the outcome and considered their dog to be fully functional with a good quality of life (Table 2) comparing to preoperative status. The functional status of the limbs was assessed and physical examination was performed. On palpation, all hips were stable at a median of 11, 5 months postoperative (negative Ortolani test) and all but one was evaluated as non-painful at limits of hip joint range of motion. We have noticed some degree of limitation during abduction maneuver, but no clinical consequences were observed. No owner noticed any abnormal position of the limb during urination. All 7 dogs had full function, where they were restored to full intended level and duration of activities without any medication. Overall, functional ability was considered excellent for all the hips, and the dogs had a normal gait.

**Table 2:** Owner responses for mid-term follow-up (6–12 months).

**#**	**Question**	**Case 1**	**Case 2**	**Case 3**	**Case 4**	**Case 5**	**Case 6**	**Case 7**
1	What is quality of life now since surgery?	Very good	Very good	Very good	Very good	Very good	Very good	Very good
2	What is willingness to play voluntarily?	Very good	Very good	Very good	Very good	Very good	Very good	Good
3	How is activity during the day?	Very good	Very good	Very good	Very good	Very good	Very good	Good
4	Stiffness at the beginning of the day?	No	No	No	No	No	Yes	No
5	Stiffness at the end of the day?	No	No	No	No	No	No	Yes
6	Indication of lameness when walking on surgery limb?	No	No	No	No	No	No	No
7	Pain when suddenly turning while walking?	No	No	No	No	No	No	No

### Complications

There were no complications noted during surgery. The most common radiographic complication reported following bilateral pelvic osteotomy in our population was pelvic canal narrowing (Figure 2d), but no clinical consequences were noted according to owner's statement. No owners reported complications related to the surgery at the time of follow-up. In one case (case 1) we suspect a superficial surgical site infection at the pubic incision after 7 days postoperative. Ten days of antibiotic administration (marbofloxacin 4 mg/kg orally once daily) resolved the complication. No major or catastrophic complications occurred during the study period. One incidence of acute onset of unilateral hind limb lameness on the operated limb was reported 3 months postoperatively (case 7). This was treated with NSAID's and resolved after 7 days.

## Discussion

The TPO 60° plate procedure resulted in subjective clinical improvement in dogs with moderate to severe hip dysplasia. Total hip replacement was not an option because of financial reasons and the femoral head and neck excision was presented as an option if TPO with 60 degree plate would generate severe complications.

Adequate healing was obtained in all of cases with a median time to final radiographic recheck of 200 days. Only a minor complication was reported without any major or catastrophic complications. Owner satisfaction and subjectively improved clinical outcomes support the use of a 60-degree TPO plate in select cases.

All cases have returned postoperatively to assess radiographic healing. The results were primary bone union and early return to full function with no secondary callus formation or degenerative joint disease. All owners were pleased with their dog's function with the dog having normal daily activities (running, and playing) or showing marked improvement after recovery from surgery (full function classification). The modification of the visual analog scale questionnaire (9) was used also in other studies (11) and we consider this subset of questions from the Hudson's evaluation to be relevant for mid- and long-term follow-up.

Regardless of manufacturer, plates typically have angles ranging from 20 to 45 degrees, with options of 20, 30, or 40 degrees being most common for triple pelvic osteotomy, whereas for double pelvic osteotomy, plates of 25 and 30 degrees are most common (5). According to our knowledge, this is the first report of 60° plate for triple pelvic osteotomy in dogs. The rationale for this angle is the our perception of better postoperative femoral head coverage and stability, although studies have demonstrated that coverage of the femoral head by the dorsal acetabular rim does not significantly increase for a 30- or 40-degree plate beyond that achieved for a 20-degree plate (2, 12). Despite these findings, an evaluation, both objective and subjective, of the inherent laxity of a given hip joint should also be considered when choosing the plate angle (5). During Ortolani tests the angles of reduction of more than 40 degrees in all dogs were subjectively assessed. Ortolani test is a subjective test, and it was difficult for us to estimate a maximum angle of reduction. This was our motivation for choosing a 60-degree TPO plate.

Although excessive ventroversion can lead to ventral luxation (11, 12), we didn't find this complication in our short series. Schrader (13) used 70 to 90 degrees acetabular fragment rotation in 77 hips of immature dogs with hip dysplasia. No ventral luxation of the femoral head was observed in the study. Anyway, he used triple pelvic osteotomy with transplantation of the greater trochanter. In his study, all hips were moderate to severely subluxated and a satisfactory functional, physical, and radiographic result was obtained in 40 of 55 hips available for long-term re-examination, which is similar to our findings.

Excessive femoral head coverage by the dorsal acetabular rim, resulting in impingement on the femoral neck, was observed after triple pelvic osteotomy with 20-degree plates (12, 14). The coverage increased in the weeks following surgery. Coverage of 82.8% ± 10.4% and Norberg angles of 121 ± 11.3 degrees were reported 6 weeks after surgery (2). In another study, the average percentage of femoral head coverage 2 months after surgery was 60.0% ± 12.1%, with Norberg angles of 109.5 ± 7.9 degrees (15). The authors consider that the increase in femoral head coverage over time following triple pelvic osteotomy may be due to postoperative increased lateral rotation associated with pelvic canal narrowing (16). Dejardin et al. (2) assessed contact area and coverage of loaded hips using serial computed tomography scan images before and after TPO. Three angles of acetabular ventroversion were studied (20, 30, and 40 degrees). They concluded that increasing acetabular ventroversion beyond 20 degrees does not significantly improve the beneficial effects of TPO and therefore should be carefully weighed against increased risks of postoperative complications associated with large angles of acetabular ventroversion. According to another study (12), based on percent coverage and Norberg angle, 20 degrees rotation of the acetabulum provides as much benefit as 30 degrees of acetabular rotation when performing a TPO. We concluded that increasing acetabular ventroversion beyond 20 degrees improves the beneficial effects of TPO without any risk of major complications associated with large angles of acetabular ventroversion.

Plates are available in both non-locking and locking styles and with a variety of screw hole configurations (5). A significantly lower incidence of screw pull-out occurs with use of locking plates compared with use of non-locking plates (17). Although only non-locking plates and cortical screws were used in our cases, no screw pull-out was observed at the final radiographic check.

While severe pelvic canal narrowing can occur following bilateral triple pelvic osteotomy procedures, particularly with greater angles of rotation, and can even lead to constipation or obstipation (2), no owner reported clinical consequences on mid-term follow-up in our cases. This is most probably due to the fact that we removed a large segment of the pubic rami (about 1 cm) during the pubic ostectomy. We think by removing a significant part of the pubic bone, there will be a less bone in the pelvis which might contribute to pelvic canal narrowing.

We consider prophylactic post-operative antibiotics to be protective against infection after TPO surgery. Similar immediate postoperative management was used in other study related to TPO surgery (17). The incisional complication was speculated because of self-trauma at the pubis incision (case 1). This was empirically treated with an additional course of antibiotics and resolved (marbofloxacin 4 mg/kg orally once daily for 10 days). No culture and sensitivity test was performed.

All cases treated originally by 60-degree TPO would have been candidates for *ab initio* THR. The reasons for selection of 60-degree TPO were related to financial constraints and an expectation of suboptimal recovery after femoral head and neck excision (FHNE) in some large breed dogs (18). The owners have chosen 60-degree TPO as a first option, knowing about the possibility of revision by FHNE in cases of inadequate recovery or complications after 60-degree TPO.

Our study has several limitations and should be taken into account when interpreting the results. The major limitation is the subjective nature of the owner postoperative assessment to evaluate the outcome. More objective methods, such as kinematic gait analysis, were not performed because of lack of adequate equipment. Our study has only few cases and it was retrospective rather than prospective and controlled. No long term-follow up was assessed which is another limitation of the study. The surgeries were performed by two surgeons (HE, CAO), and here may have been some surgeon variation in technique. The aim of our study was to report radiographic and clinical findings and we did not statistically analyze data or established control groups. Radiographic assessment of osteoarthritis is important in evaluating mid- and long-term surgical outcome, but it was beyond the objective of this study and further research is necessary.

We achieved a favorable subjective mid-term owner-assessed outcome in this small retrospective case series of 9 hips. Subjective assessment of adequate radiographic healing at a mean of 200 days after surgery provides important support of the 60-degree TPO technique. We feel that this technique can be successfully performed and is a viable option in the treatment plan for dogs with hip dysplasia.

## Data Availability Statement

The original contributions presented in the study are included in the article/supplementary material, further inquiries can be directed to the corresponding author/s.

## Ethics Statement

The animal study was reviewed and approved by Ethics Committee of University of Agricultural Sciences and Veterinary Medicine Cluj-Napoca. Written informed consent was obtained from the owners for the participation of their animals in this study.

## Author Contributions

HE, CO, and AT contributed to the design of the study and performed the surgeries, radiographic and clinical examination. CY, WM, and JM participated in the subsequent discussions and revisions of the entire text. All authors contributed to the article and approved the submitted version.

## Conflict of Interest

The authors declare that the research was conducted in the absence of any commercial or financial relationships that could be construed as a potential conflict of interest.
